# Reconstitution of Mdm2-Dependent Post-Translational Modifications of p53 in Yeast

**DOI:** 10.1371/journal.pone.0001507

**Published:** 2008-01-30

**Authors:** Barbara Di Ventura, Charlotta Funaya, Claude Antony, Michael Knop, Luis Serrano

**Affiliations:** European Molecular Biology Laboratory (EMBL), Heidelberg, Germany; IBM Thomas J. Watson Research Center, United States of America

## Abstract

p53 mediates cell cycle arrest or apoptosis in response to DNA damage. Its activity is subject to a tight regulation involving a multitude of post-translational modifications. The plethora of functional protein interactions of p53 at present precludes a clear understanding of regulatory principles in the p53 signaling network. To circumvent this complexity, we studied here the minimal requirements for functionally relevant p53 post-translational modifications by expressing human p53 together with its best characterized modifier Mdm2 in budding yeast. We find that expression of the human p53-Mdm2 module in yeast is sufficient to faithfully recapitulate key aspects of p53 regulation in higher eukaryotes, such as Mdm2-dependent targeting of p53 for degradation, sumoylation at lysine 386 and further regulation of this process by p14^ARF^. Interestingly, sumoylation is necessary for the recruitment of p53-Mdm2 complexes to yeast nuclear bodies morphologically akin to human PML bodies. These results suggest a novel role for Mdm2 as well as for p53 sumoylation in the recruitment of p53 to nuclear bodies. The reductionist yeast model that was established and validated in this study will now allow to incrementally study simplified parts of the intricate p53 network, thus helping elucidate the core mechanisms of p53 regulation as well as test novel strategies to counteract p53 malfunctions.

## Introduction

Acting as a sensor of DNA damage, p53 dictates life or death depending on the ability of the cell to cope with the damage and repair it. It is therefore subject to a tight regulation achieved by a complex protein network [1]. p53, an evolutionarily conserved protein from worm to humans, is a sequence-specific DNA-binding transcription factor. Under normal growth conditions, p53 is kept latent in various ways, *e.g*. by keeping it in the cytoplasm, by targeting it for proteasomal degradation, or by inhibiting binding to its DNA target sites [2]. When exposed to DNA-damage, the cell invokes p53 to take action. p53 is quickly stabilized and its transcriptional activity is strongly enhanced as a result of the nuclear accumulation of the protein and modifications within both its N- and C-termini [2]. Although the rise in p53 protein levels is partially due to a higher translational rate of p53 mRNA, the major players in p53 stabilization are post-translational modifications, which prevent the association of p53 with its negative regulators [3]. Mdm2 is the best-characterized p53 negative regulator to date. Mdm2 N-terminus binds the N-terminal transactivation domain of p53 [4]. This binding inhibits p53 transcriptional activity independently of the ubiquitin ligase function of Mdm2, which resides in the C-terminal RING-finger domain [5]. In addition to mono- and poly-ubiquitylation, Mdm2 is involved in the neddylation and sumoylation of p53 [6], [7]. While neddylation has been reported to inhibit p53 transcriptional activity [7], the precise role of sumoylation on the activity and localization of p53 is still unclear, although p53 sumoylation seems to be related to the association of the protein with nuclear organelles such as the nucleolus [6], [8] and PML bodies [8].

Given the high complexity of the p53 network, distinguishing between the contributions of each individual protein to p53 regulation is a difficult task. We aimed at studying p53-Mdm2 functional interplay in the absence of the other regulators in the p53 network, while at the same time maintaining important eukaryotic cell characteristics. We have chosen to use the budding yeast *Saccharomyces cerevisiae* since p53 and the majority of its regulators are foreign to its genome, therefore this organism provides us with the perfect tool to study p53-Mdm2 interaction “in isolation” but at the same time in a cellular context. In the past it has been shown that p53 can bind to its consensus sequences and recruit the yeast transcription machinery to start expression of downstream genes [9]–[11], a process that can be inhibited by co-expression with Mdm2 [4]. Here we show that Mdm2 can interact with endogenous yeast pathways to ubiquitylate and sumoylate p53. Ubiquitylation leads to p53 degradation, while sumoylation is essential for the localization of p53-Mdm2 complexes to yeast nuclear bodies. These domains are morphologically akin to human PML bodies and human PML proteins are also found to relocate there. We conclude that yeast can be used as a model organism to study p53 post-translational modifications and their regulatory effects on p53 function and localization.

## Materials and Methods

### Yeast strains, growth media and growth conditions

The yeast strain used in this study is ESM356-1 (S288c genetic yeast background), with genotype Mat**a** ura3-53 leu2Δ1 his3Δ200 trp1Δ63. Cultures were grown at 30°C in synthetic complete (SC) media with 2% raffinose, lacking one or more amino acids for maintenance of plasmids. Basic yeast techniques were as described in [12].

### Plasmids

The human p53 wild type cDNA was amplified by PCR from the plasmid pRS314-SN (kind gift of Prof. Bert Vogelstein) and cloned into the centromeric plasmid p413-GALS, containing the HIS3 marker, the GALS promoter and the CYC1 terminator [13]. For microscopy analysis, the p53 cDNA into pRS314-SN was substituted with the fusion p53-eCFP generated by PCR as in [14]. The human mdm2 wild type cDNA was amplified by PCR from pCDNA3.1-Mdm2 (kind gift of Dr. Cayetano Gonzales) and cloned into the centromeric plasmids p416-TEF/GALS containing the URA3 marker, either the TEF or GALS promoter and the CYC1 terminator. For microscopy analysis, the mdm2 cDNA was substituted with the fusion mdm2-eYFP generated by PCR as in [14]. The human p14^ARF^ cDNA was digested out of the plasmid pLPC (kind gift of Dr. Cayetano Gonzales) and cloned into the vector p414-TEF, containing the TRP1 marker, the TEF promoter and the CYC1 terminator. The ECFP and EYFP genes were amplified from the plasmids pECFP-N1 and pEYFP-N1 respectively (Clontech). The human mdm2 cDNA lacking the RING finger domain was generated inserting a STOP codon in place of residue 440, using the Quickchange site-directed mutagenesis (Stratagene). All p53 and Mdm2 mutants used in this study were obtained by Quickchange site-directed mutagenesis (Stratagene). All constructs used in this study have been fully sequenced.

### Analysis of protein turnover in yeast cells

Cultures were cultivated at 30°C on a shaker at 230 rpm for 14 hours and diluted in the morning to an OD_600_ of 0.1–0.2. Two hours later (with cells typically at OD_600_ of 0.3), galactose was added to a final concentration of 0.5%. Cells were incubated for 1.5 hours before glucose was added to a final concentration of 3% to stop gene expression. The first sample (sample ‘0’) was collected half an hour after addition of glucose to the medium. The remaining samples were collected two, four and six hours afterwards. Crude cell extracts for western blotting were generated as described (Knop et al. 1999). Equal volumes of samples were analysed by SDS-PAGE followed by Western Blotting. p53 was detected using the monoclonal antibody Pab 1801 (Santa Cruz biotech). Mdm2 was detected using the monoclonal antibody SMP14 (Santa Cruz biotech). PGK was detected using the monoclonal antibody 22C5 (Molecular Probes). The secondary antibody was peroxidase-conjugated donkey anti-mouse (Jackson Immuno Reasearch Laboratories). Protein levels were quantified using Adobe Photoshop calculating ratios to the loading control (PGK).

### Purification of 6xHIS-tagged proteins for detecting SUMO-conjugated p53

1l yeast culture was grown and induced (at OD∼1.5) for 2.5 hours with galactose at a final concentration of 2% and then lysed mechanically using glass beads, under denaturing conditions. Lysates were then purified on Ni^+^-NTA sepharose beads (GE Healthcare). Elution was done with 200 mM imidazole. Detection of sumoylated proteins was done using a rabbit polyclonal antibody anti-Smt3 [15]. Detection of p14^ARF^ was done using the rabbit polyclonal antibody ab3642 (abcam).

### Microscopic techniques

For live cells imaging, cells were adhered with Poly-lysine on small glass bottom Petri dishes (MaTek). All live cell experiments were performed at RT. Live cell imaging was performed on an imaging system (DeltaVision Spectris; Applied Precision) equipped with filters (Chroma Technology Corp.) to detect CFP, YFP and red fluorescent proteins, a 100x NA 1.4 oil immersion objective (plan Apo, IX70; Olympus), softWorRx software (Applied Precision), and a CoolSNAP HQ camera. ImageJ software was used to mount the images and to produce merged colour images. The width of the stacks taken was always such that it encompassed at least the thickness of the cell nucleus (spacing 0.5 µm). Maximum projections of the fluorescence images were generated and coloured using ImageJ software. For the time-lapse microscopy, p53 localization over time was not imaged to avoid bleaching of the signals. Only Mdm2 localization was followed over time. p53 expression in each cell was verified taking stacks before and after the time-lapse. For immunofluorescence microscopy, cells were fixed with 3.7% formaldehyde for 60 min in the medium (adjusted to 0.1 M K_x_H_y_PO4, pH 6.5). Digestion of cells using Zymolyase 100T and immunolabeling was performed as described in [16]. For triple labeling, primary antibodies (p53: goat polyclonal N-19 (Santa Cruz biotech); Mdm2: rabbit polyclonal H-221 (Santa Cruz biotech); NOP1: mouse monoclonal, kind gift of Dr. Ed Hurt) were detected using Cy2-, Cy3-, and Cy5-labeled donkey antibodies (Jackson Immuno Reasearch Laboratories).

### Electron microscopy

Yeast cells were cryofixed in an EMPACT-2 high-pressure freezer (Leica Microsystems, Vienna, Austria) and further processed for freeze substitution. The freeze substitution solution was 0.2 % Glutaraldehyde, 0.1 % Uranyl acetate and 1 % water in acetone. The samples were then embedded in lowicryl HM20 (Polysciences, Warrington, PA, USA) in a freeze substitution device (EM-AFS Leica Microsystems, Vienna, Austria). Ultrathin sections were prepared using a Reichert UltracutE microtome (Leica Microsystems, Vienna, Austria). Non-specific binding sites were blocked by incubating sections in blocking buffer (0.1 % fish skin gelatin, 1.5 % BSA in PBS) for 15 min. Sections were incubated with mouse primary antibodies (either anti-p53 Pab 1801 or anti-Mdm2 SMP14, concentration 1∶2) for 30 min before extensive rinsing and further incubation with a rabbit anti-mouse (DAKO cytomation, concentration 1∶150) linker antibody followed by final Protein A-Gold (10 nm, CMC university medical center Utrecht) conjugates for 20 min. Sections were fixed in 1% glutaraldehyde in PBS for 5 min, rinsed with water and contrasted with uranyl acetate and lead citrate. Sections were then viewed and pictures were acquired in a CM120 biotwin electron microscope (FEI, Eindoven, The Netherlands) operating at 100 kV. Digital acquisitions were made with a Keen View CCD camera (Soft Imaging System, Muenster, Germany).

## Results

Yeast has been first used to show the ability of Mdm2 to bind to and conceal the p53 transactivation domain [4]. It has been then used to study p53 transcriptional activity [9], as well as the requirements for p53 degradation in human cells by E6-E6AP [21]. These results proved that yeast expressing human p53 and Mdm2 can grow, that p53 and Mdm2 can interact in yeast and that p53 can bind to consensus sequences, recruit the yeast transcription machinery and induce expression of downstream genes, as we also observed with Mdm2 (Figure S1). Mdm2-dependent p53 post-translational modifications, such as ubiquitylation and sumoylation, and subsequent p53 degradation have not yet been shown to occur in this organism. The conservation of the ubiquitin–and ubiquitin-like–pathway from yeast to humans gives us good reasons to believe that the human E3 ubiquitin ligase Mdm2 could interact with the yeast ortologue(s) of the human E2 conjugating enzyme(s) that allow the ubiquitylation reaction to take place [22]. Indeed, the high sequence similarity of the interested proteins suggests the conservation of their binding and functional activities. We therefore devised an experimental set-up to study Mdm2-dependent p53 post-translational modifications in yeast.

### p53 is stable in yeast and localizes to the nucleus

**Figure 1:**
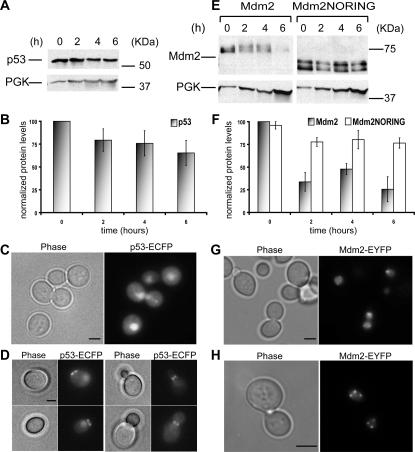
Stability and localization of the human p53 and Mdm2 proteins in yeast. (A) Total yeast cell extracts from exponentially growing cells were immunoblotted for p53 over time. 3-phosphoglycerate kinase (PGK) was used as loading control. (B) Quantifications of p53 protein levels are shown. Data represent the mean ± standard error (SE), for *n = 3* independent experiments. (C,D) Maximum projections from fluorescence image stacks of live yeast cells expressing p53-ECFP were obtained with a DeltaVision workstation. Bar, 2 µm. *E,F,G,H.* Same as in *(A,B,C,D)* but for Mdm2.

We expressed p53 under the control of the inducible GAL1 promoter. Expression of the gene was triggered by the addition of galactose to the medium and repressed adding glucose to the same medium. The pool of protein made in the time window between addition of galactose and glucose was then followed for a period of six hours. p53 showed little degradation in yeast (around 30% in six hours) under these conditions (Figures 1A and 1B).

We also performed *in vivo* microscopy on cells expressing the fusion protein p53-ECFP, which has been previously shown to be functional and to retain a wild type localization in human cells [14]. p53 accumulated in the yeast nucleus and was also present in the cytoplasm (Figure 1C), which corresponds to the reported localization in human cells. In addition, in budding cells, the protein was also found at the bud neck and sometimes unbudded cells showed a ring-like localization at the plasma membrane, which might correspond to sites of previous cytokinesis (Figure 1D).

### Mdm2 is degraded in yeast and localizes to nuclear speckles

We studied Mdm2 degradation following the same method used for p53. We observed that, unlike p53, Mdm2 was rapidly degraded in yeast (Figures 1E and 1F) (around 60% in two hours). Mutating histidine 452 in the RING finger domain of Mdm2 was not sufficient to abolish its E3 ligase activity in yeast (data not shown). When a truncated protein missing the C-terminal RING finger domain (Mdm2NORING) was used instead of the wild type protein, we found that degradation was strongly impaired (Figures 1E and 1F) (around 25% in six hours). Since the RING finger domain has been shown to be responsible for Mdm2 self-ubiquitylation in human cells (Fang et al. 2000), our data suggest that Mdm2 is degraded in yeast due to self-ubiquitylation.

The fusion protein Mdm2-EYFP [14] was detected in the nucleus of yeast living cells (Figure 1 G), often within speckles (Figure 1 F).

### p53 is degraded in yeast in the presence of Mdm2

**Figure 2:**
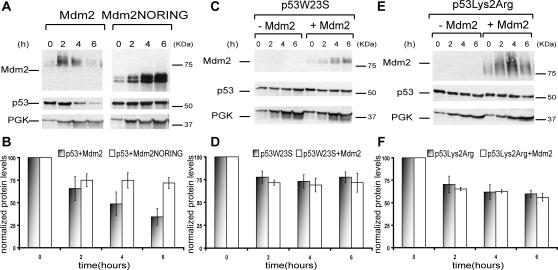
Mdm2 ubiquitylates p53 in yeast leading to p53 degradation. (A) Total yeast cell extracts from exponentially growing cells were immunoblotted for the indicated proteins over time. 3-phosphoglycerate kinase (PGK) was used as loading control. (B) Quantifications of protein levels are shown. Data represent the mean ± standard error (SE), for *n = 3* independent experiments. (C,D) as in *(A,B)* but for p53W23S. (E,F) as in *(A,B)* but for p53Lys2Arg.

We then co-expressed p53 and Mdm2. While the mdm2 cDNA was under the control of a constitutive promoter (TEF), the p53 cDNA was under the control of the inducible GAL1 promoter. In this way, Mdm2 proteins were constantly produced, while only a pool of p53 proteins was produced which we followed over time. p53 was degraded in the presence of Mdm2 (Figures 2A and 2B). When a truncated Mdm2 protein (Mdm2NORING) missing the RING finger domain–shown to be responsible for Mdm2 E3 ubiquitin ligase activity in human cells (Fang et al. 2000)–was used instead of wild type Mdm2, p53 showed little degradation (Figures 2A and 2B), as in the absence of Mdm2 (Figures 1A and 1B). Similarly, degradation was severely reduced when we co-expressed Mdm2 with either a mutant p53 with impaired binding to it (p53W23S) (17) (Figures 2C and 2D) or with a mutant p53 in which the C-terminal lysines at positions 372, 373, 382 and 383 were replaced by arginines (p53Lys2Arg), to prevent ubiquitylation keeping the overall charge of the protein unchanged [18] (Figures 2E and 2F). Taken together, these data suggest that Mdm2 ubiquitylates p53 targeting it for degradation in yeast.

### p53 and Mdm2 co-localize to PML-like nuclear bodies in yeast

**Figure 3:**
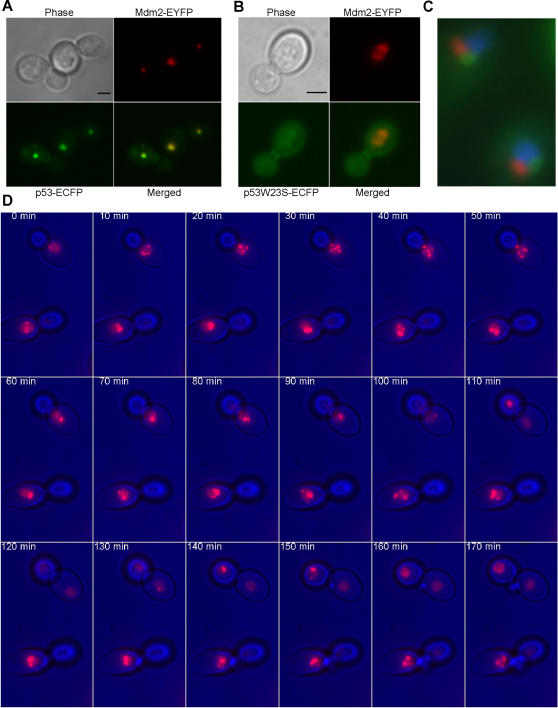
p53 and Mdm2 co-localize in yeast cells. (A,B) Maximum projections from fluorescence image stacks of live yeast cells expressing the indicated fusion proteins were obtained with a DeltaVision workstation. Bar, 2 µm. (C) Indirect immunofluorescence of fixed yeast cells stained for p53-Mdm2 bodies (primary antibody: goat polyclonal N-19 anti-p53; secondary antibody: Cy2-labeled donkey anti-goat; showed in green), Nop1 (primary antibody: mouse monoclonal; secondary antibody: Cy5-labeled donkey anti-mouse; showed in red) and chromatin (detected with Höechst, showed in blue). (D) Time-lapse microscopy on yeast cells expressing p53 and Mdm2 obtained recording image stacks and then performing maximum projections of live yeast cells at the indicated times. Galactose was added directly into the Petri dish to trigger p53 expression and time-lapse started shortly after. Mdm2 is shown in red and the phase-contrast image of the cells in blue.

**Figure 4:**
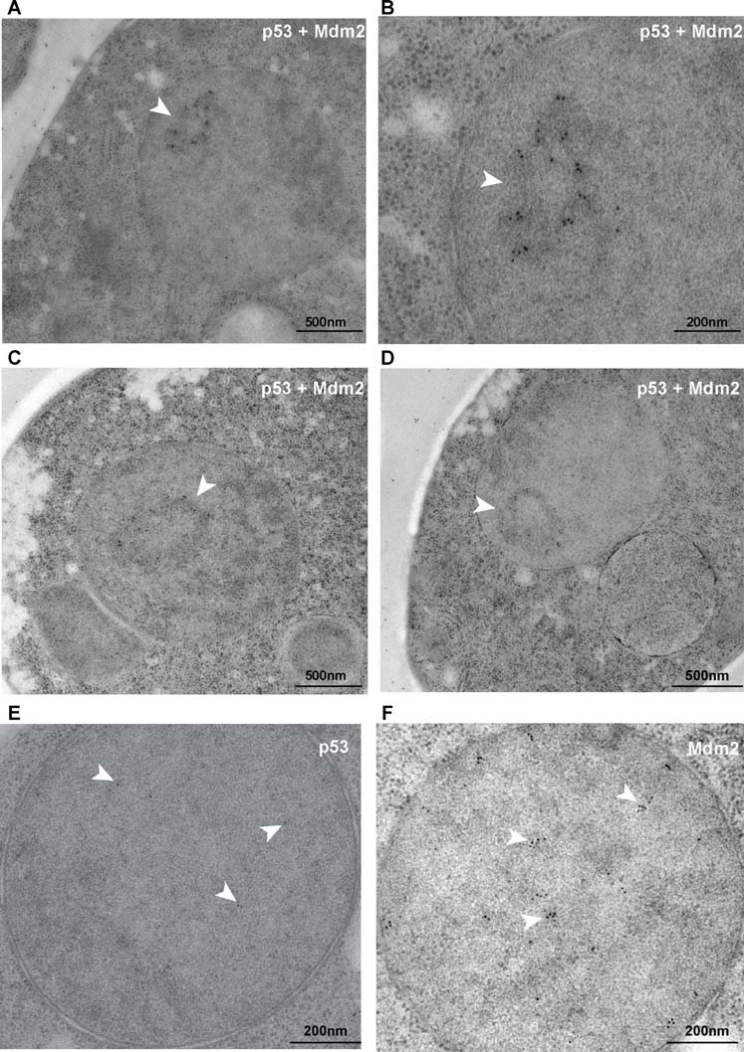
p53-Mdm2 complexes localize to yeast nuclear bodies morphologically akin to human PML bodies. Immunogold labeling against p53 (A,D,E) or Mdm2 (C,F) of thin sections of yeast cells expressing the indicated proteins. Total number of cells analyzed for each case: 10. (B) Magnification of the p53-Mdm2 body (arrowhead) in the cell in panel A.

Notably, co-expressing p53-ECFP and Mdm2-EYFP revealed that the two proteins co-localized to one or more nuclear dots, to which we will refer as p53-Mdm2 bodies (Figure 3A). This co-localization was lost when the mutant p53W23S was used in place of wild type p53 (Figure 3B), indicating that direct binding of the proteins was required for this localization. These structures were visible also when performing indirect immunofluorescence (Figure 3C), therefore they were not triggered by fusing the proteins to the fluorescent tags. Using an antibody against the yeast nucleolar protein Nop1, we analyzed the localization of the p53-Mdm2 bodies in respect to the nucleolus. We did not find substantial overlap between the signals corresponding respectively to the anti-Nop1 and the anti-p53/Mdm2 antibodies, therefore we concluded that p53-Mdm2 bodies did not reside in the nucleolus (Figure 3C). The p53-Mdm2 bodies were most often found in regions of the nucleus adjacent to the nucleolus (Figures 3C and 4A,C,D). Time-lapse microscopy revealed that p53-Mdm2 co-localization is stable over time (Figure 3D). Soon after its expression, p53 is found in the discrete loci where Mdm2 is localized (data not shown), but the p53-Mdm2 complexes then move towards a bigger structure (Figure 3D). We next investigated the morphology and localization within the cell of the p53-Mdm2 bodies using immunoelectron microscopy (Figure 4). The immunostaining detected areas containing electron dense fibrillar spheroids with a doughnut-like shape (13 out of 16 cells analyzed, Figures 4A–D), reminiscent of human PML bodies [19]. Cells expressing only p53 or only Mdm2 showed a diffuse pattern of the gold particles (Figures 4E and 4F), therefore confirming that the formation of the bodies required both p53 and Mdm2.

### PML can re-locate to the p53-Mdm2 bodies in yeast

**Figure 5:**
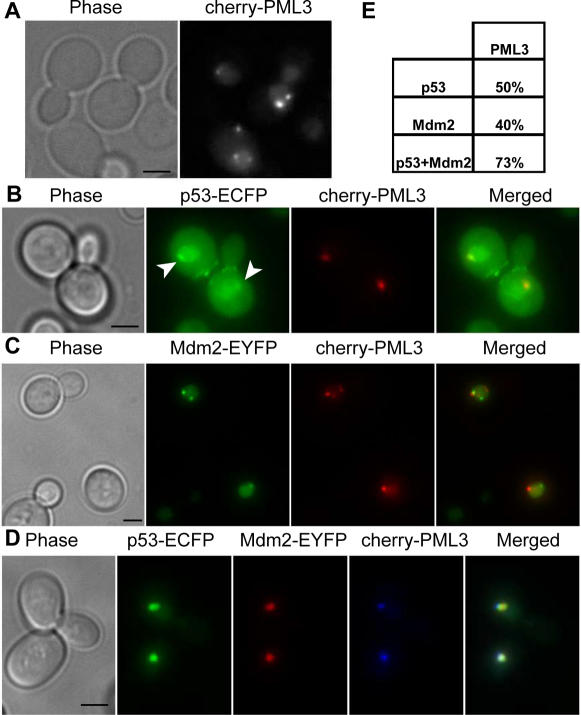
The human PML protein can relocate to the p53-Mdm2 bodies in yeast. (A,B,C,D) Maximum projections from fluorescence image stacks of live yeast cells expressing the indicated fusion proteins were obtained with a DeltaVision workstation. Bar, 2 µm. (E) Table showing the percentage of cells in which the indicated proteins co-localize. Percentages were calculated from more than 100 cells each.

Given the morphological similarity between the p53-Mdm2 bodies in yeast and human PML bodies, we next investigated the localization of human PML, alone or together with p53 and/or Mdm2 in yeast cells. PML localized to the nucleus, mostly to one or more discrete loci (Figure 5A). Upon co-expression with PML, p53 could still accumulate in the nucleus (Figure S2) and co-localized with PML in 50% of the cells to relatively large, but still discrete subregions of the nucleus (Figures 5B and 5E). However, unlike when it was co-expressed with Mdm2, p53 was still detectable in the nucleoplasm and the cytoplasm (compare Figures 3A and 5B). When co-expressed, Mdm2 and PML co-localized in 40% of the cells, indicating that the two proteins could interact but also retained separate localizations (Figures 5C and 5E). When p53, Mdm2 and PML were co-expressed, we observed the typical localization of p53-Mdm2 complexes to the bodies, into which PML could relocate in 73% of the cases (Figures 5D and 5E).

### Mdm2 sumoylates p53 in yeast alone or synergistically with p14^ARF^

**Figure 6:**
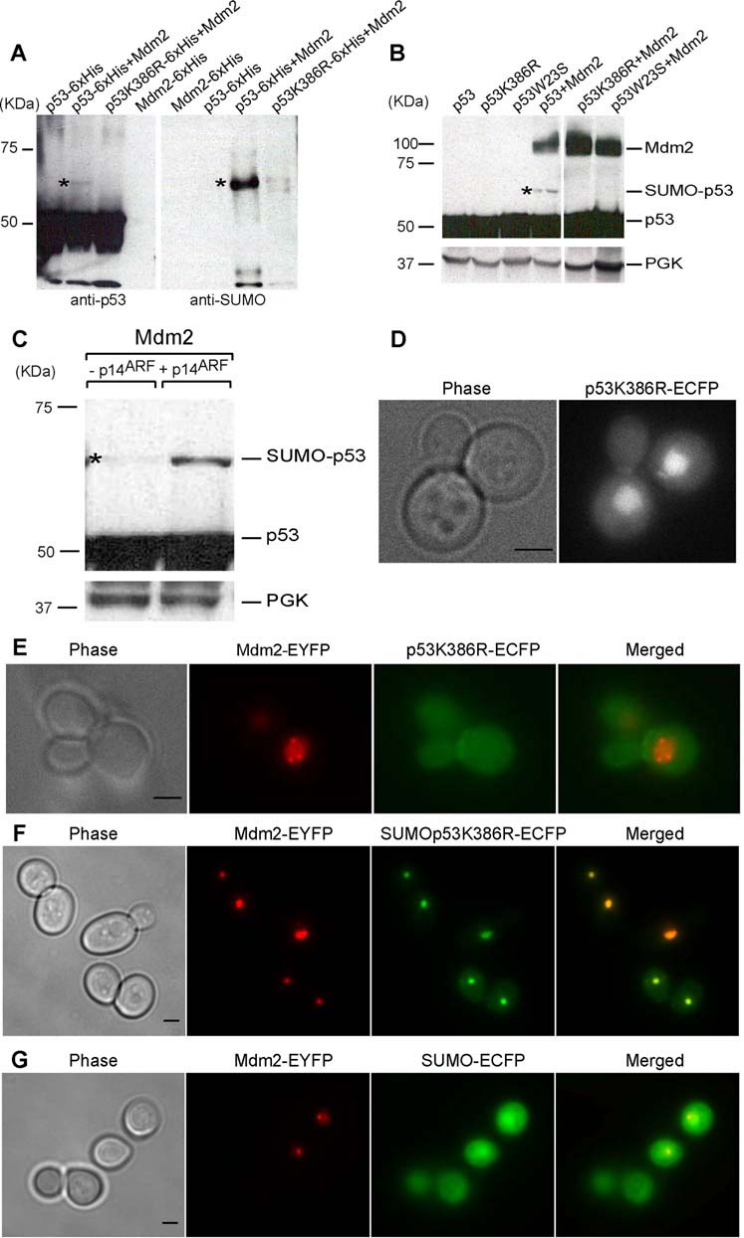
Mdm2 sumoylates p53 in yeast on lysine 386 and sumoylation is required for p53-Mdm2 complexes recruitment to yeast nuclear bodies. (A) His-tagged proteins were purified from cell extracts and analyzed by immunoblotting using anti-p53 and anti-Smt3 antibodies. (B,C) Sumoylated p53 (asterisks) as detected by immunoblotting on crude yeast lysates. An irrelevant lane was cut off as indicated by a space. PGK immunoblotting served as loading control. (D–G) Maximum projections from fluorescence image stacks of live yeast cells expressing the indicated fusion proteins were obtained with a DeltaVision workstation. Bar, 2 µm.

We affinity precipitated p53 from denaturing extracts of cells expressing the protein either alone or with Mdm2. p53 precipitates were then analyzed for sumoylation using an anti-Smt3 antibody, which is directed against the yeast SUMO protein (Figure 6A). Only in cells co-expressing wild type p53 and Mdm2 we could detect a band which was simultaneously recognized by the anti-p53 and the anti-Smt3 antibodies, and which ran at a size consistent with the conjugation of a single SUMO protein to p53 (Figure 6A, asterisks). This slower-migrating form of p53 could also be detected on western blot on crude lysates, provided high amount of proteins was loaded on the gel and the film was exposed for longer times (Figures 6B and S3).

In human cells, lysine 386 is known to be the major site for p53 sumoylation since, when this residue is mutated to arginine, p53 sumoylation is abolished (Gostissa et al. 1999). When we used this mutant p53K386R in yeast in place of wild type p53 no SUMO conjugation was detectable on p53 (Figures 6A,B and S3). Given that binding of p53 to Mdm2 is required for the modification to take place, sumoylation was also abolished when we co-expressed Mdm2 with a mutant p53 which is impaired in binding to Mdm2 (p53W23S, see above) (Figures 6B and S3).

Since p14^ARF^ has been shown to enhance Mdm2-dependent p53 sumoylation in human cells (6), we co-expressed p53, Mdm2 and p14^ARF^ in yeast cells. We found that indeed p14^ARF^ led to enhanced p53 sumoylation (Figure 6C).

### p53 sumoylation and Mdm2 are necessary for p53-Mdm2 co-localization to the nuclear bodies

Sumoylation is known to affect the subcellular localization of proteins, often correlating with their affiliation with nuclear bodies [8], [20]. Consequently, we thought that SUMO conjugation to p53 might be related to the localization of p53-Mdm2 complexes to nuclear bodies in yeast. We found that, when expressed alone, p53K386R could accumulate in the nucleus like wild type p53 (Figure 6D), but indeed inhibiting sumoylation disrupted p53 and Mdm2 co-localization to the nuclear domain (Figure 6E). In light of this result we can also interpret the failed co-localization of p53W23S with Mdm2 (Figure 3B) since binding of p53 to Mdm2 is necessary for the sumoylation to take place (see above and Figures 5B and S3). When SUMO was fused to the non-sumoylatable p53 mutant p53K386R (SUMOp53K386R-ECFP) we could rescue the formation of the bodies, provided Mdm2 was present in the same cells (Figure 6F). These data suggest that the interaction with Mdm2 is required for the localization to the bodies and that SUMO has a binding rather than a structural role in this localization. Finally, SUMO itself did not localize to nuclear bodies with Mdm2 (Figure 6G).

## Discussion

The aim of this study was to reconstitute a minimal part of the complex p53 network in yeast to study the mechanisms of p53 regulation by MDM2 in isolation from their natural genomic and proteomic environments. By expressing human p53 and Mdm2 in normally growing yeast cells we could reproduce Mdm2-dependent p53 degradation, which is the key outcome of Mdm2 negative regulation on p53. Even if we did not manage to directly detect ubiquitins attached to p53, our data strongly support the idea that Mdm2 could interact with the yeast enzymes in the ubiquitylation pathway to ubiquitylate p53 and target it to proteasomal degradation.

Recently, the central role of Mdm2 in keeping p53 levels low under normal growth conditions has been questioned, due to the discovery of several other E3 ubiquitin ligases for p53, some of which are not, unlike Mdm2, p53 transcriptional targets [23]. Our findings suggest that Mdm2 is a key negative regulator for p53 even in normal growth conditions.

Besides ubiquitylation, Mdm2 has also been reported to sumoylate and neddylate p53 [6], [7], but its precise role in these processes is less well understood. Our findings here indicate that Mdm2-mediated p53 sumoylation is an important event that takes place in yeast on the same lysine residue targeted in human cells. Moreover, the mechanisms of sumoylation could be reconstituted in yeast since, when the human protein p14^ARF^ was co-expressed with p53 and Mdm2, we observed an increase in p53 sumoylation levels in accordance with what was reported by Chen and colleagues [6].

Taken together, these results suggest that p53 ubiquitylation and sumoylation by Mdm2 and its increased activation by p14^ARF^ do not require additional components.

p53 degradation was not detectable when samples were collected from cells close to the stationary phase (data not shown). An intriguing possibility is that the enzymes in the ubiquitylation pathway needed for p53 degradation are somehow inactive at that stage. Interestingly, Mdm2 levels also decreased (Figure 2A) despite its being under a constitutive promoter. This suggests that the ubiquitylation reaction towards p53 somehow enhances Mdm2 self-ubiquitylation, and in fact when the protein was co-expressed with a mutant p53 which cannot bind to it (p53W23S), Mdm2 levels accumulated over time (Figure 2C).

Unexpectedly, p53-Mdm2 complexes localize to nuclear bodies in yeast, which exhibit a doughnut-like shape morphologically akin to human PML bodies [19]. Human PML proteins expressed in yeast can relocate to these domains. Beyond the morphological similarity between the PML and p53-Mdm2 bodies, we find that sumoylation of p53 is essential for this localization, just like sumoylation of PML is essential for the PML bodies' formation [24]. When co-expressed with Mdm2, the non-sumoylatable p53K386R can no longer localize to nuclear bodies and fails to accumulate in the nucleus (Figures 6D and 6E). We suggest that sumoylation might affect the equilibrium between p53 nuclear import and export helping to retain p53 into the nucleus. Sumoylation is also needed to localize p53 to the nuclear bodies, therefore, when sumoylation is impaired, p53 export is less counterbalanced and we see an accumulation of the protein in the cytoplasm. Binding to Mdm2 also helps retaining p53 into the nucleus since, when the binding is abolished, p53 also accumulates in the cytoplasm (Figure 3B). p53-Mdm2 co-localization to the PML-like bodies is recovered when SUMO is fused to p53K386R N-terminus, indicating a binding rather than a structural role for SUMO in this process. In human cells, sumoylation does not seem to be necessary for p53 recruitment into PML bodies, since p53K386R is also found there [25]. The discrepancy between what is observed in yeast and human cells could lie in the existence of so far unidentified human proteins that can relocate the non-sumoylatable p53 to PML bodies (beyond PML itself) and that are absent in yeast. Since PML has been shown to be necessary for the formation of human PML bodies [24], our data hint at the existence of a yeast protein(s) with no straightforward sequence similarity to PML but with likely structural and/or functional homology to it. Finally, p53 recruitment to PML bodies has been shown to take place via direct binding to PML [25]. Our findings reveal that only p53-Mdm2 complexes are localized to the bodies, while p53 alone, co-expressed with PML, co-localizes with PML but is also found in the cytoplasm and the nucleoplasm (Figure 5B). We therefore speculate an active role for Mdm2 in the recruitment of p53 in human PML bodies.

Our study of the human p53-Mdm2 module in yeast gives rise to a whole new set of issues which might be important for understanding p53 biology in higher eukaryotes, for instance Mdm2 localization to nuclear speckles similar to that of telomere-binding proteins such as RAP1 (Laroche et al. 2000), p53 localization to the septin ring, Mdm2 ability to recruit sumoylated p53 to yeast nuclear bodies which resemble human PML bodies. Importantly, we have now set the basis for further reconstruct the p53-Mdm2 network in yeast. This should allow dissecting the network and studying its dynamical aspects such as the ability of the p53-Mdm2 negative feedback loop to generate oscillations.

## Supporting Information

Figure S1 p53-dependent Mdm2 expression in yeast cells. Cells carrying a plasmid with the p53 cDNA under the GAL promoter and a plasmid with the Mdm2 cDNA under a p53-responsive promoter were grown up to exponential growth phase. Induction of p53 expression was done with 0.5% final concentration of galactose (corresponding to time 0 min). Glucose was not added to stop induction.
(0.62 MB TIF)

Figure S2 Co-expression with PML does not inhibit accumulation of p53 into the nucleus. (A,B,C) Maximum projections from fluorescence image stacks of live yeast cells expressing the indicated fusion proteins were obtained with a DeltaVision workstation. Bar, 2 µm. (A) Same as in Figure 1C.
(3.79 MB TIF)

Figure S3 Mdm2 sumoylates p53 in yeast on lysine 386. Sumoylated p53 (asterisks) as detected by immunoblotting on crude yeast lysates using only anti-p53 antibody. The samples are the same as those shown in Figure 6B. An irrelevant lane was cut off as indicated by a space. PGK immunoblotting served as loading control.
(0.34 MB TIF)
