# Supplementary figures and images for: Reconstitution of Mdm2-Dependent Post-Translational Modifications of p53 in Yeast
Source: PLoS One. 2008 Jan 30;3(1):e1507. doi: 10.1371/journal.pone.0001507 (PMC2200829; doi:10.1371/journal.pone.0001507)

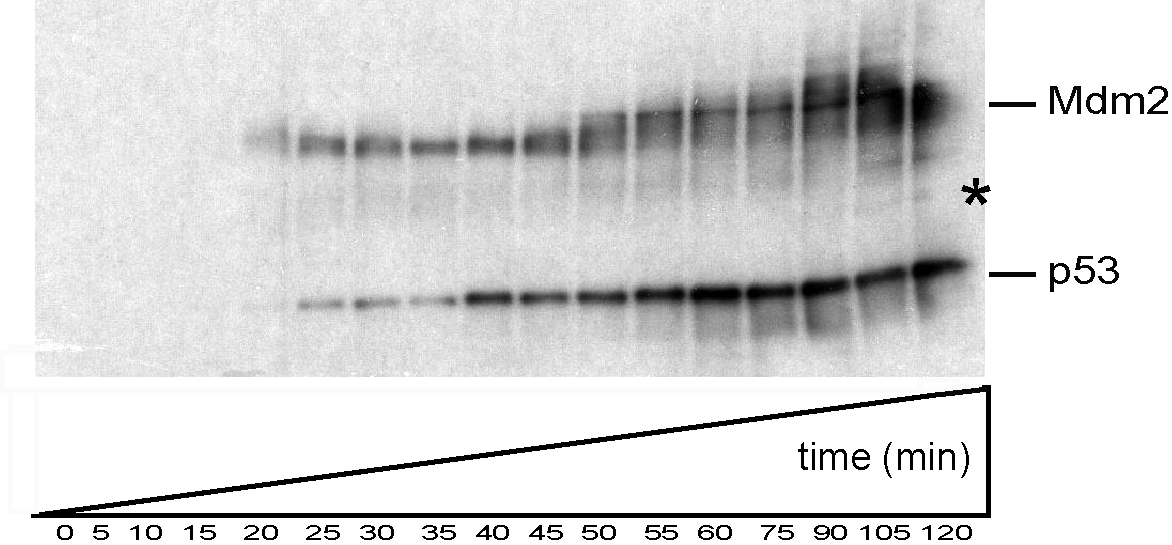

Supplement: Figure S1 — p53-dependent Mdm2 expression in yeast cells. Cells carrying a plasmid with the p53 cDNA under the GAL promoter and a plasmid with the Mdm2 cDNA under a p53-responsive promoter were grown up to exponential growth phase. Induction of p53 expression was done with 0.5% final concentration of galactose (corresponding to time 0 min). Glucose was not added to stop induction. (0.62 MB TIF) [file pone.0001507.s001.tif]

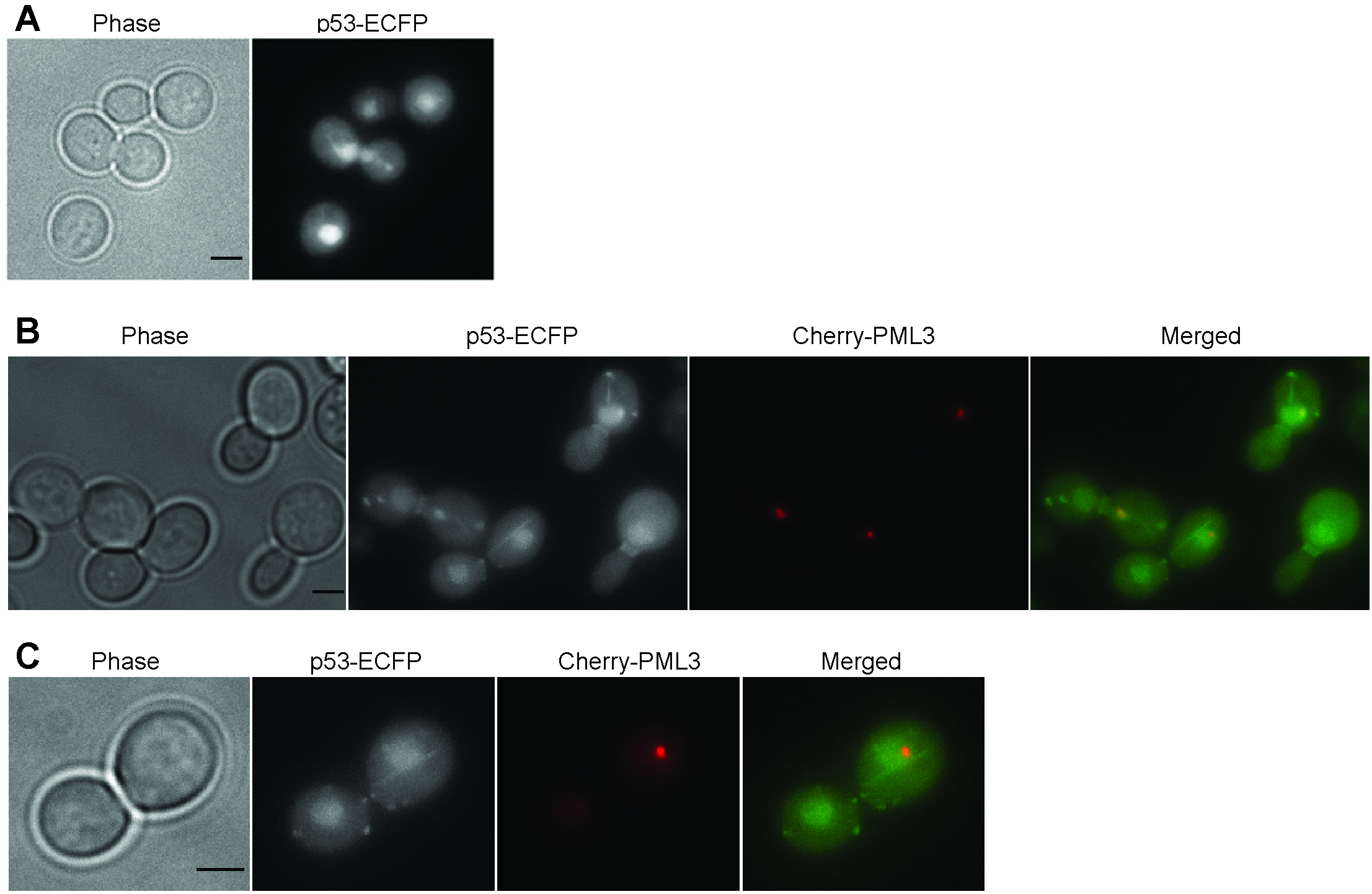

Supplement: Figure S2 — Co-expression with PML does not inhibit accumulation of p53 into the nucleus. (A,B,C) Maximum projections from fluorescence image stacks of live yeast cells expressing the indicated fusion proteins were obtained with a DeltaVision workstation. Bar, 2 µm. (A) Same as in Figure 1C. (3.79 MB TIF) [file pone.0001507.s002.tif]

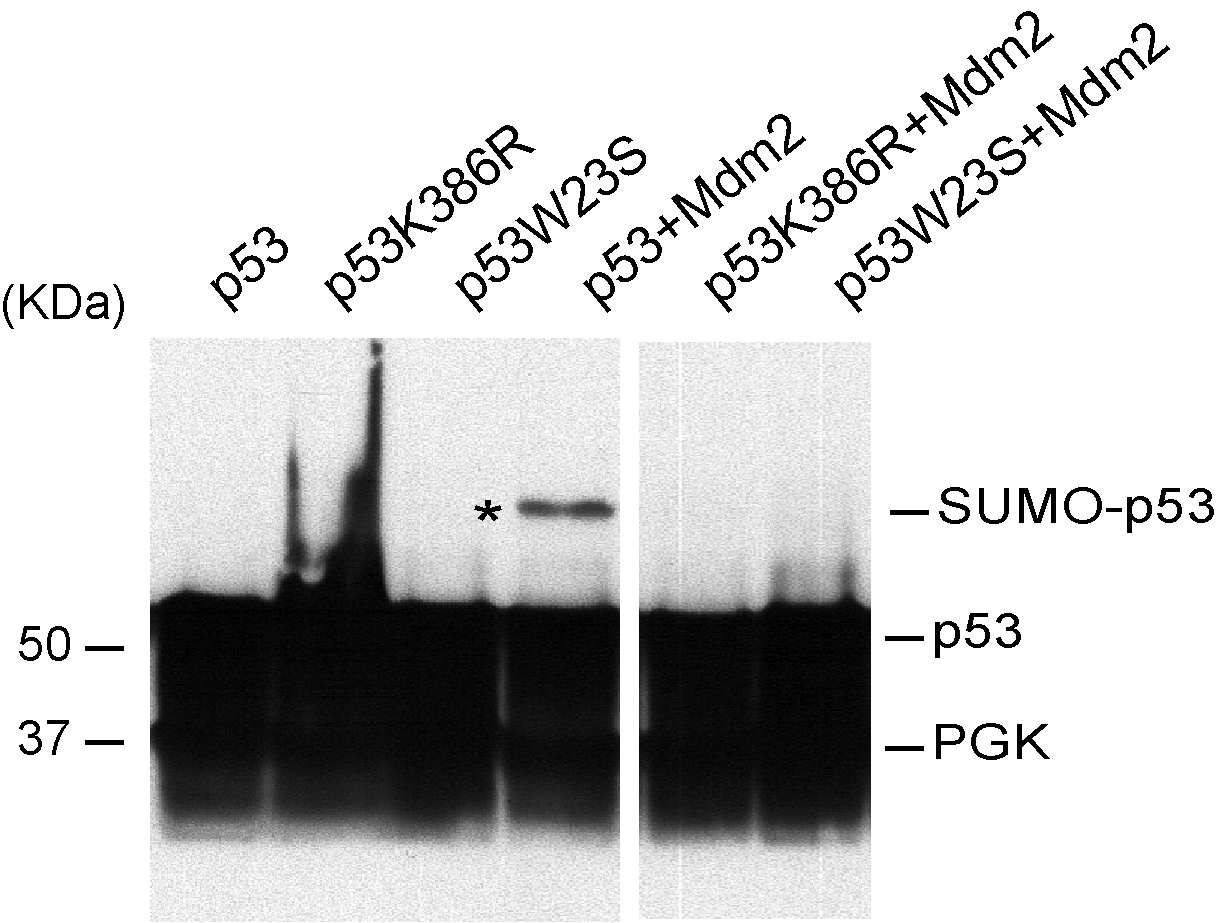

Supplement: Figure S3 — Mdm2 sumoylates p53 in yeast on lysine 386. Sumoylated p53 (asterisks) as detected by immunoblotting on crude yeast lysates using only anti-p53 antibody. The samples are the same as those shown in Figure 6B. An irrelevant lane was cut off as indicated by a space. PGK immunoblotting served as loading control. (0.34 MB TIF) [file pone.0001507.s003.tif]
